# MRC-5 fibroblast-conditioned medium influences multiple pathways regulating invasion, migration, proliferation, and apoptosis in hepatocellular carcinoma

**DOI:** 10.1186/s12967-015-0588-8

**Published:** 2015-07-22

**Authors:** Songming Ding, Guoliang Chen, Wu Zhang, Chunyang Xing, Xiao Xu, Haiyang Xie, Aili Lu, Kangjie Chen, Haijun Guo, Zhigang Ren, Shusen Zheng, Lin Zhou

**Affiliations:** Division of Hepatobiliary and Pancreatic Surgery, Department of Surgery, First Affiliated Hospital, Zhejiang University School of Medicine, Hangzhou, Zhejiang People’s Republic of China; Division of Oncology Department, First Affiliated Hospital, Zhejiang University School of Medicine, Hangzhou, Zhejiang People’s Republic of China; Key Laboratory of Combined Multi-Organ Transplantation, Ministry of Public Health, Hangzhou, Zhejiang China; Key Laboratory of Organ Trans-Plantation, Zhejiang Province, Hangzhou, Zhejiang China

**Keywords:** MRC-5-conditioned medium, Non-classical epithelial–mesenchymal transition, Integrin/FAK pathway, EMT-related transcription factors

## Abstract

**Background:**

Carcinoma associated fibroblasts (CAFs), an important component of tumor microenvironment, are capable of enhancing tumor cells invasion and migration through initiation of epithelial–mesenchymal transition (EMT). MRC-5 fibroblasts are one of the CAFs expressing alpha-smooth muscle actin. It is ascertained that medium conditioned by MRC-5 fibroblasts stimulate motility and invasion of breast cancer cells. However, its role in hepatocellular carcinoma (HCC) is less clear. The aim of our study was to investigate the effect of MRC-5-CM on HCC and explore the underlying mechanisms.

**Methods and results:**

Using a combination of techniques, the role of MRC-5-CM in HCC was evaluated. We determined that MRC-5-CM induced the non-classical EMT in Bel-7402 and MHCC-LM3 cell lines. Initiation of the non-classical EMT was mainly via quintessential redistribution of α-, β- and γ-catenin, P120 catenin, E-cadherin, and N-cadherin, rather than up-regulation of typical EMT-related transcription factors (i.e., Snail, Twist1, ZEB-1 and ZEB2). We also found that MRC-5-CM potentiated both the migration and invasion of Bel-7402 and MHCC-LM3 cells in mesenchymal movement mode through activation of the α6, β3, β4, β7 integrin/FAK pathway and upregulation of MMP2. The flow cytometric analysis showed that MRC-5-CM induced G1 phase arrest in Bel-7402 cells with a concomitant reduction of S phase cells. In contrast, MRC-5-CM induced S phase arrest in MHCC-LM3 cells with a concomitant reduction of cells in the G2/M phase. MRC-5-CM also inhibited apoptosis in Bel-7402 cells while inducing apoptosis in MHCC-LM3 cells.

**Conclusion:**

Collectively, MRC-5-CM promoted HCC cell motility and invasiveness through initiation of the non-classical EMT, including redistribution of α-, β- and γ-catenin, P120 catenin, E-cadherin, and N-cadherin, activation of the integrin/FAK pathway, and upregulation of MMP2. Hence, MRC-5-CM exerted distinct roles in Bel-7402 and MHCC-LM3 cell viability by regulating cyclins, cyclin dependent kinases (CDKs), CDK inhibitors (CKIs), Bcl-2 family proteins and other unknown mechanosensors.

**Electronic supplementary material:**

The online version of this article (doi:10.1186/s12967-015-0588-8) contains supplementary material, which is available to authorized users.

## Background

Hepatocellular carcinoma (HCC) ranks as the fifth most common cancer in men (554,000 cases, 7.5% of the total) and the ninth in women (228,000 cases, 3.4%) [1]. Despite advancements in early diagnosis and surgical treatment over the last few decades, HCC remains the third most common cause of cancer-related death worldwide [2, 3]. The aggressive nature of HCC is, by and large, attributed to vascular invasion and resistance to apoptosis [4, 5]. Hence, the current pressing matter is identification of the crucial pathways linked to HCC invasion/metastasis and apoptosis/proliferation.

The epithelial–mesenchymal transition (EMT) has been suggested to be involved in the progression of various cancers, including HCC [6]. Tumor cells that have undergone the EMT exhibit increased invasive/metastatic and apoptosis-resistant properties, among others. Several pathways capable of inducing the EMT have been identified [6–8] and the roles of transcription factors (i.e., Snail, Twist1, ZEB-1 and ZEB-2) in EMT modulation have also been evaluated [9–12]. However, the exact mechanism of the EMT remains incompletely understood and currently appears as the tip of the iceberg. Recent reports have highlighted that integrins and laminins as the main components of the cell adhesion functional unit [13], and these molecules are often correlated with cancer progression by potentiating the EMT via cooperation with other signaling effectors [14, 15]. Therefore, there is a compelling need to distinguish the specific roles of laminins and integrins in HCC.

The influence of conditioned medium from MRC-5 fibroblast on breast cancer cell motility and invasion potential has been well addressed [16]. However, the exact mechanisms remain elusive. In this study, the effect of MRC-5 fibroblast-conditioned medium (MRC-5-CM) on HCC cell proliferation, apoptosis, cell motility and invasion was examined. Further, we evaluated the expression profiles of cell cycle- and apoptosis-associated proteins, EMT-related proteins, matrix metalloproteases (MMPs), laminins, integrins, and other adhesion molecules to explore there associated molecular mechanisms in HCC cells. These findings will be useful for the identification of potential targets for therapeutic intervention of HCC.

## Methods

### Cell culture

The human lung fibroblast cell line MRC-5 was a generous gift from Dr. Xi Chen (Zhejiang University, China). Bel-7402 and MHCC-LM3 cell lines were purchased from Shanghai Cell Bank, Chinese Academy of Sciences. MRC-5 cells were maintained in RPMI-1640 media (Gibco) supplemented with 10% fetal bovine serum (FBS; Sigma-Aldrich) at 37°C in a 5% CO_2_ water-saturated environment. Conditioned medium of MRC-5 cells was collected as follows. Cells were cultured until 70–90% confluency, at which point the used medium was collected and passed through a 0.22-µm filter, diluted at a 1:1 ratio with RPMI-1640 containing 10% FBS. RPMI-1640 medium supplemented with 10% FBS serverd as the control medium. Bel-7402 and MHCC-LM3 cells were respectively cultured in the conditioned medium from MRC-5 cells for 21 days (n = 3). Bel-7402 was subcultured once a week at a ratio of 1:3 or 1:5. MRC-5 and MHCC-LM3 were subcultured once a week at a ratio of 1:1 or 1:2. 5 ml MRC-5-CM was used when HCC cells were cultured in 25 cm^2^ cell culture flasks. 20 ml MRC-5-CM was used when HCC cells were cultured in 75 cm^2^ cell culture flasks.

### Transwell assay

The migration and invasion assays were performed using Transwell chambers with 8 µm pore filters (Millipore, Billerica, MA, USA). The filters used for invasion assays were coated with 30 µl pre-diluted Matrigel (diluted at a ratio of 1:8 with serum-free RPMI-1640 medium) (BD Bioscience, San Jose, CA, USA). 5 × 10^4^ cells in 0.3 ml serum-free RPMI-1640 medium were added to the upper chambers. Then, 0.8 ml RPMI-1640 medium supplemented with 10% FBS was added to the lower chambers as a chemoattractant. After incubation for 48 h, cells on the upper membrane surface were wiped off, and the cells that invaded across the Matrigel membrane were washed with phosphate-buffered saline (PBS), fixed with 100% methanol, rinsed in PBS, and stained with 0.2% crystal violet. The invaded cells were counted (5 randomly chosen high-power fields for each membrane) under a light microscope (at 200× magnification). The filters used for migration assays were not coated with Matrigel. All experiments were performed at least in triplicate.

### Western-blot analysis

Whole cells were lysed on ice in a lysis buffer (RIPA, Beyotime, Shanghai, China) with a protease inhibitor mixture cocktail (Roche, Switzerland) after culturing in MRC-5-CM for 21 days. After centrifugation at 12,000 rpm for 30 min at 4°C, the protein concentrations of supernatants in samples were measured by the BCA protein assay (Thermo scientific, Rockford, IL, USA). Equal amounts of protein (50 µg) were separated by 10–12% NUPAGE Bis–Tris Gel (Invitrogen, CA, USA) electrophoresis (constant voltage: 120 mv) and transferred onto polyvinylidene fluoride (PVDF, 0.45 µm) membranes (constant current: 350 mA for 70/120 min). After being blocked by Tris-buffered saline and Tween 20 (TBST) buffer containing 5% non-fat powder milk for 2 h, the membranes were incubated with primary antibodies overnight on ice. After washing the membranes several times in TBST while agitating, detection was performed using the appropriate secondary HRP-conjugated anti-mouse or anti-rabbit antibody. Immunoreactive bands on the blots were visualized with enhanced chemiluminescence reagent ECL kit (Beit Haemek, Israel) and intensities were quantified using Glyko BandScan 5.1 software. Anti-α, β and γ-catenin, anti-E-cadherin, anti-ZO-1, anti-N-cadherin, anti-Fibronectin, anti-ZEB-1, ZEB-2, Snail, and Twsit1 and anti-vimentin primary antibodies were purchased from (Abcam); anti-MMP-1, 2, 3, 11, 12, 13, 14, 17 and 21, anti-p53, anti Annexin IV, anti-Vitronectin, anti-Ezrin, anti-P120, anti-Laminin A1, Laminin B3, anti-Integrin A6, B1, B3, B4 and B7, anti-FAK, P-FAK-Y397, Src and P-Src-Y529 primary antibodies were purchased from (Epitomics); anti-Cdc-2, P-Cdc-2-Tyr15, CDK4, CDK6, Cyclin A, Cyclin D1, Cyclin D3, Cyclin E2, P15, P16, P21,P27, Rb, P-Rb-S811, P-Rb-S795, P-Rb-S780; anti-Bcl-2, P-Bcl-2-Thr56, Bcl-xl, Bad, P-Bad-Ser112, Bak, Bax, Bik, Mcl-1, Puma and anti-β-actin were from (Cell Signaling Technology).

### Confocal immunofluorescent analysis

5 × 10^5^ cells were implanted onto a cell culture dish for 24 h (NEST Biotech, Hong Kong, China) after culturing in MRC-5-CM for 21 days. Confocal immunofluorescent analysis: cells were fixed with paraformaldehyde for 30 min, then permeabilized with 0.1% Triton X-100 for 10 min at room temperature, and thereafter sealed with goat serum for 1 h at room temperature following primary antibodies incubation in the dark for 24 h at 4°C. Washed three times with PBS and then cells were incubated with Alexa Flour^®^ 488 IgG donkey anti-mouse or anti-rabbit second antibodies (1:300, Invitrogen, USA) in the dark for 1 h at room temperature. Then, nuclei were stained with propidium iodide for 5 min. Fluorescence images were photographed with confocal microscopy (Leica DMIRE2, Germany) (at 10 × 63 magnification).

## Flow cytometry

For cell cycle analysis, the cells were fixed with ice-cold 75% ethylalcohol at 4°C overnight and incubated with propidium iodide (BD Bioscience, CA) at 4°C in the dark for 30 min. For apoptosis analysis, cells were incubated with Annexin V-FITC (BD Bioscience, CA) and propidium iodide for 15 min at 4°C in the dark. After staining, the cells were analyzed using a flow cytometer (CYTOMICS FC 500, Beckman Coulter, Miami, FL, USA). All experiments were performed at least in triplicate.

### Statistical analysis

Independent Student t test was used to analyze the differences between 2 groups using SPSS 16.0 software (SPSS, Chicago, IL, USA). Statistical significance was accepted if p < 0.05.

## Results

### Cell morphology and cell motility

**Figure 1:**
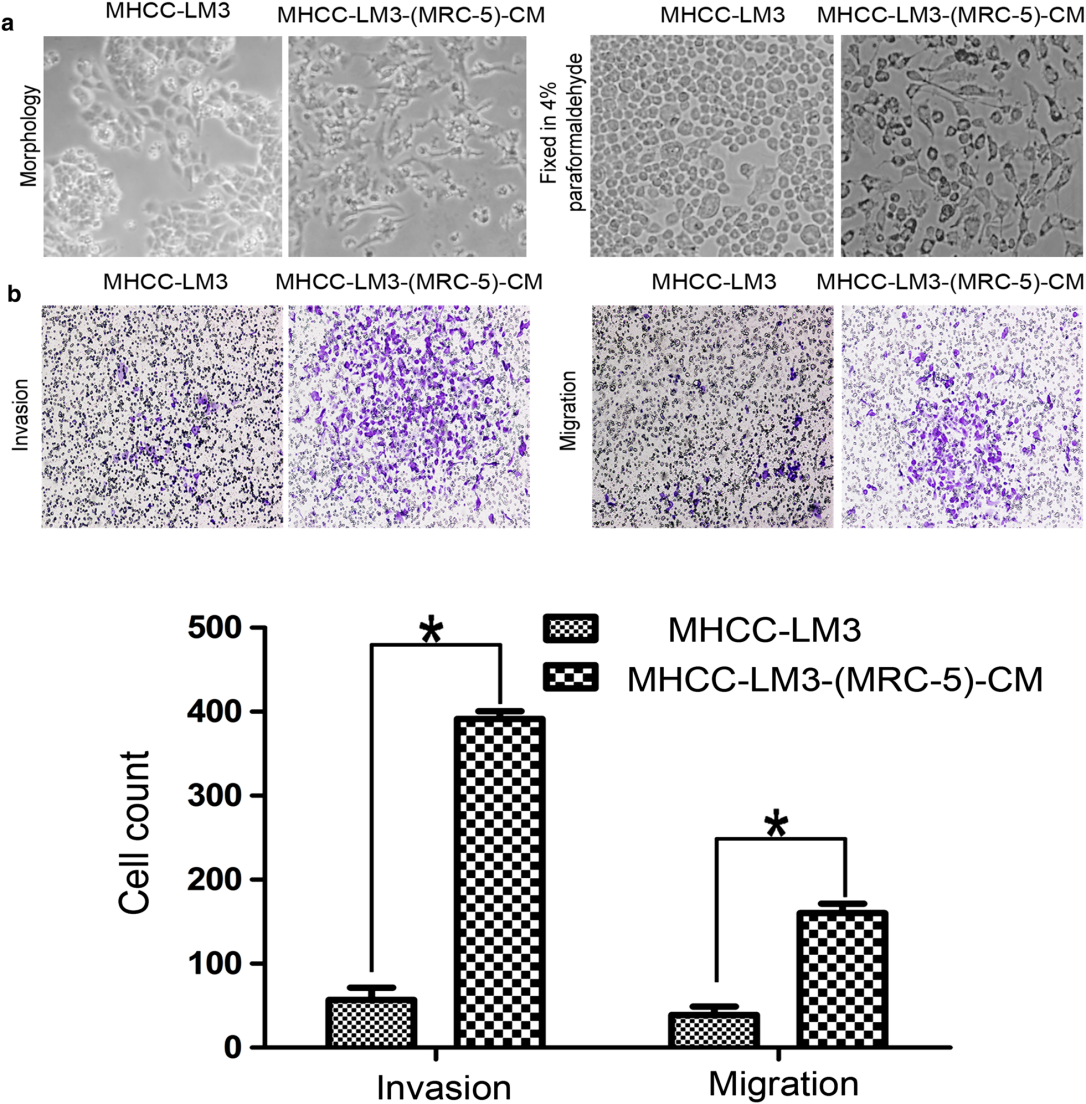
Alterations in cell morphology and motility. **a** Morphological changes in MHCC-LM3 cells after culture in MRC-5-CM. **b** Transwell assays are shown: histograms represent cell invasion and migration over 72 h. Invasion and migration of MHCC-LM3 cells cultured in MRC-5-CM for 21 days were increased relative to the control (*P* < 0.05).

Bel-7402 cells were cultured in MRC-5-CM, hereafter referred to as Bel-7402-(MRC-5)-CM. MHCC-LM3 cells were cultured in MRC-5-CM, hereafter referred to as MHCC-LM3-(MRC-5)-CM. After culturing in MRC-5-CM for 21 days, Bel-7402-(MRC-5)-CM cells became elongated and spread-out. The cell motility and invasiveness potentials were significantly augmented compared with control (the results have been previously published). Similarly, after culturing in MRC-5-CM for 21 days, MHCC-LM3-(MRC-5)-CM cells were extended and broad elaborate cell protrusions were observed. The cell motility and invasiveness potentials were also significantly enhanced (*P* < 0.05, Figure 1).

### Expression of EMT-related proteins, laminins and integrins

**Figure 2:**
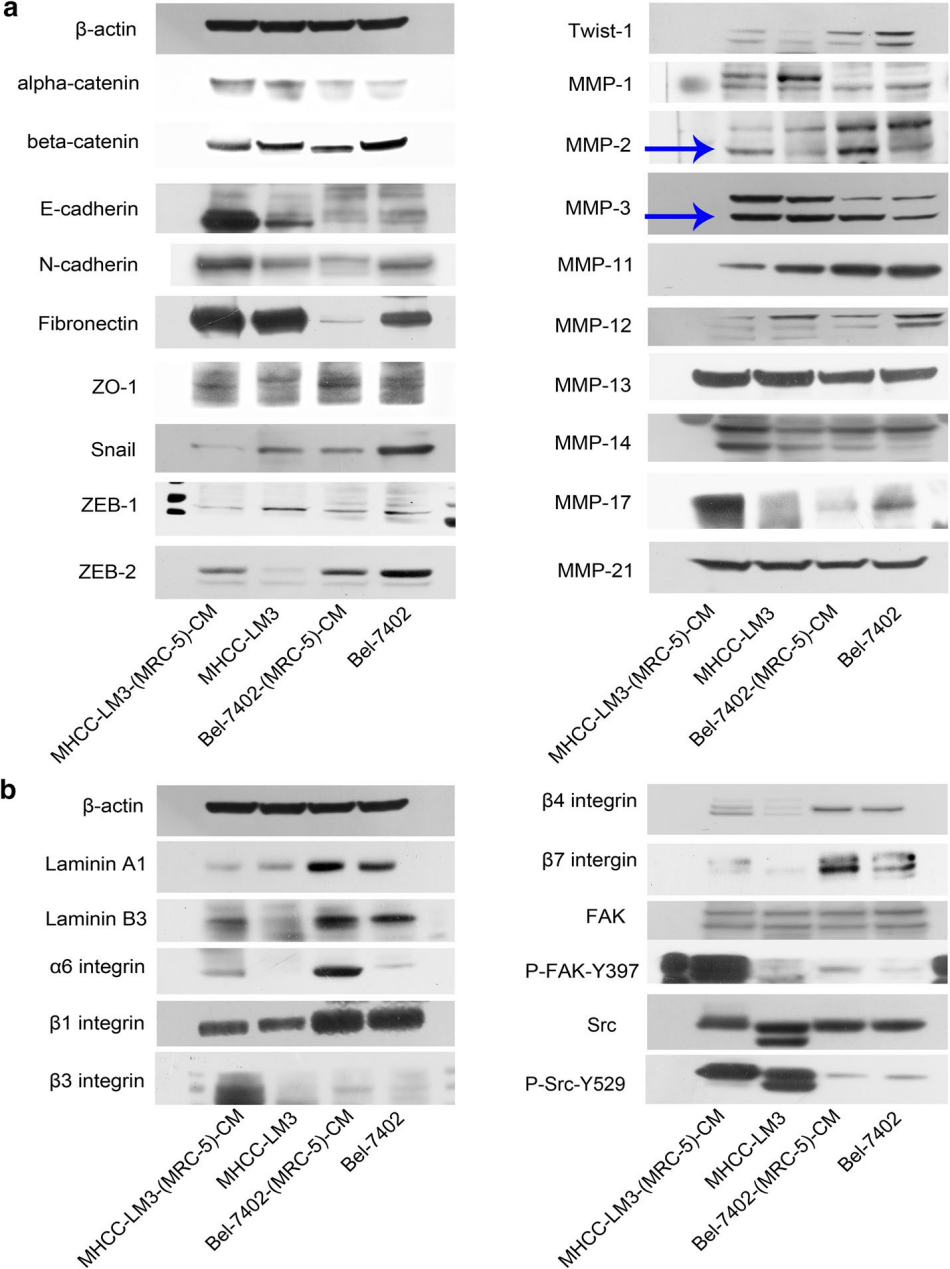
Protein expression alterations in Bel-7402 and MHCC-LM3 cells cultured in MRC-5-CM for 21 days. **a** The expression profiles of epithelial markers, mesenchymal markers and MMPs. **b** The expression profiles laminins, integrins, FAK and Src.

The EMT has been implicated in human cancer metastasis. To determine the mechanism of enhanced invasion and migration of Bel-7402-(MRC-5)-CM and MHCC-LM3-(MRC-5)-CM, the expression profiles of epithelial markers E-cadherin, α-catenin and ZO-1; mesenchymal markers N-cadherin, β-catenin and Fibronectin; and EMT-promoting transcription factors Snail, Twsit1, ZEB-1, and ZEB-2 and MMP-1, -2, -3, -11, -12, -13, -14, -17, -21 were evaluated by Western-blot analysis (Figure 2a). Mesenchymal markers Snail, Twist1, ZEB-1, ZEB-2, N-cadherin, β-catenin and fibronectin were reduced in Bel-7402-(MRC-5)-CM compared with the control (Bel-7402). The epithelial marker ZO-1 was increased. However, α-catenin remained unchanged. Furthermore, E-cadherin was downregulated. The expression profiles of MMPs showed that the expressions of MMP-2 and -3 were markedly upregulated in Bel-7402-(MRC-5)-CM, whereas MMP-1, -11, -13, -14 and -21 remained unchanged. Conversely, MMP-12 and -17 were reduced.

The expression of EMT-related proteins was also evaluated in MHCC-LM3 and in MHCC-LM3-(MRC-5)-CM (Figure 2a). Mesenchymal markers Twist1, ZEB-2, and N-cadherin were increased in MHCC-LM3-(MRC-5)-CM compared with the control (MHCC-LM3). However, Snail, ZEB-1 and β-catenin were reduced, while fibronectin remained unchanged. Moreover, epithelial markers ZO-1 and E-cadherin were increased. The expression profiles of MMPs showed that MMP-2, -14 and MMP-17 were increased in MHCC-LM3-(MRC-5)-CM. MMP-3, -13 and -21 remained unchanged. Conversely, MMP-1, -11 and -12 were reduced. Therefore, enhancement of invasion and migration of Bel-7402-(MRC-5)-CM and MHCC-LM3-(MRC-5)-CM was not via a canonical EMT program. This contradictory phenomenon suggests the counterintuitive hypothesis in which MRC-5-CM-stimulated cell migration and invasion in the HCC cells occurs via a non-classical EMT pathway.

Laminins and integrins are intimately correlated with cancer invasion and metastasis. Therefore, the expression of laminins, integrins and associated signaling molecules were evaluated in Bel-7402, Bel-7402-(MRC-5)-CM, MHCC-LM3 and in MHCC-LM3-(MRC-5)-CM (Figure 2b). We concluded that α6 and β3 integrins were upregulated in Bel-7402-(MRC-5)-CM and in MHCC-LM3-(MRC-5)-CM relative to the control. β7 integrin was significantly increased in Bel-7402-(MRC-5)-CM and moderately increased in MHCC-LM3-(MRC-5)-CM. β4 integrin was increased significantly in MHCC-LM3-(MRC-5)-CM and moderately increased in Bel-7402-(MRC-5)-CM. Laminin A1 was upregulated only in Bel-7402-(MRC-5)-CM, while laminin B3 was upregulated only in MHCC-LM3-(MRC-5)-CM. β1 integrin remained unchanged. P-FAK-Y397 was significantly up-regulated in both Bel-7402-(MRC-5)-CM and MHCC-LM3-(MRC-5)-CM. Src remained unchanged in Bel-7402-(MRC-5)-CM but was silenced in MHCC-LM3-(MRC-5)-CM. P-Src-Y529 which inactivates Src, was reduced in Bel-7402-(MRC-5)-CM but increased in MHCC-LM3-(MRC-5)-CM. Collectively, these data indicate that Bel-7402-(MRC-5)-CM and MHCC-LM3-(MRC-5)-CM increase in cell motility through elevated expression of laminins, integrins, the activated form of FAK and other downstream signaling molecules (the ratio discrepancy was listed in Additional files 1, 2, 3, 4: Figure S1–4).

### Redistribution of epithelial and mesenchymal markers and cell motility-associated adhesion molecules

**Figure 3:**
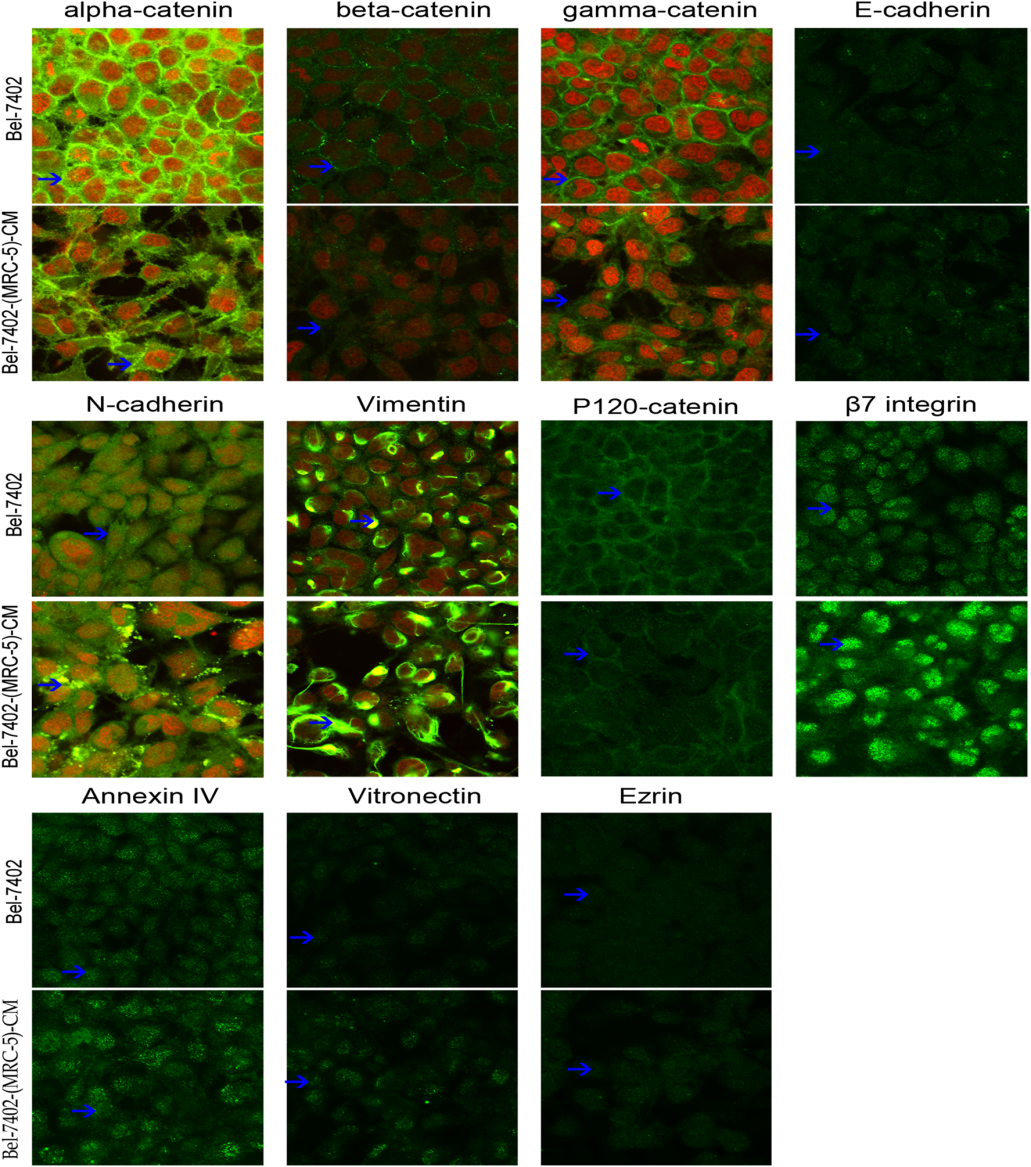
Immunofluorescence analysis of epithelial markers, mesenchymal markers and cell motility-associated adhesion molecules in Bel-7402 cells compared with Bel-7402-(MRC-5)-CM. *Green fluorescence* represents staining of the corresponding protein; *red fluorescence* represents nuclear DNA staining by DAPI. Fluorescence images were captured using a confocal microscope (at 10 × 63 magnification).

**Figure 4:**
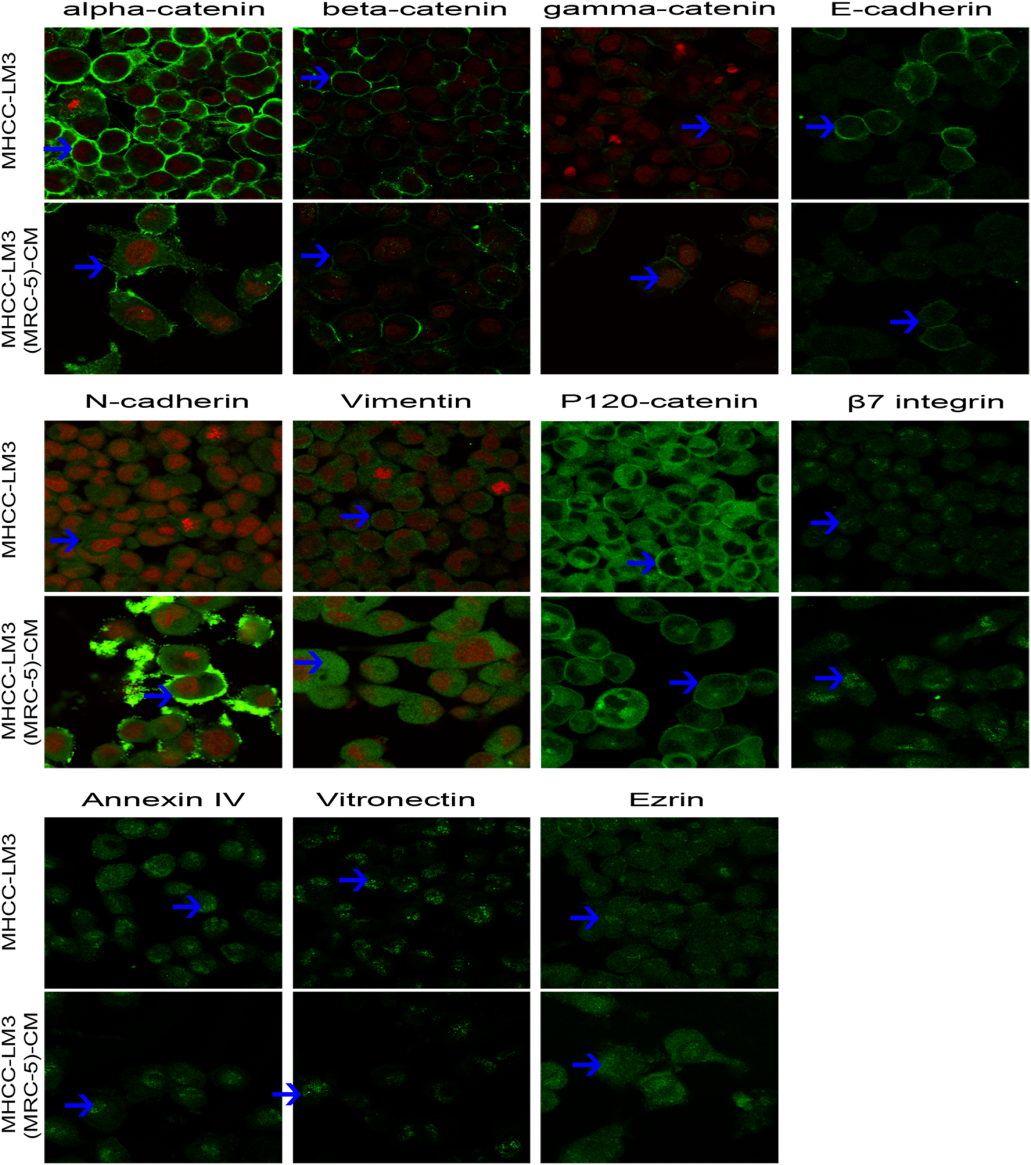
Immunofluorescence analysis of epithelial markers, mesenchymal markers and cell motility-associated adhesion molecules in MHCC-LM3 cells compared with MHCC-LM3-(MRC-5)-CM. *Green fluorescence* represents staining of the corresponding protein; *red fluorescence* represents nuclear DNA staining by DAPI. Fluorescence images were captured using a confocal microscope (at 10 × 63 magnification).

To further explore the mechanisms of enhanced cell invasion and migration in Bel-7402-(MRC-5)-CM and MHCC-LM3-(MRC-5)-CM, immunofluorescence analysis was performed. The expression of α-, β- and γ-catenin and P120 catenin on cell membrane were significantly reduced in Bel-7402-(MRC-5)-CM relative to the control, and N-cadherin was distributed in a disorderly fashion on Bel-7402-(MRC-5)-CM cell membranes. However, reduced membrane expression of E-cadherin in Bel-7402-(MRC-5)-CM was not observed, the basic membrane expression of E-cadherin in Bel-7402 is relatively low, which could explain this observation. Vimentin and β7 integrin were increased dramatically, while Ezrin (which serves as an intermediate between the plasma membrane and the actin cytoskeleton) was decreased moderately. Vitronectin which promotes cell spreading and migration through specific interactions with integrins, was upregulated slightly. Annexin IV (which is implicated in a range of cellular functions such membrane aggregation, actin configuration, and signaling) was up-regulated slightly (Figure 3). These results also revealed that membrane expression of α-, β- and γ-catenin, P120 catenin and E-cadherin were reduced in MHCC-LM3-(MRC-5)-CM compared with the control, and N-cadherin was localized in a disorderly fashion on the MHCC-LM3-(MRC-5)-CM cell membrane. The results also showed that γ-catenin in MHCC-LM3-(MRC-5)-CM had an increasing tendency to translocate to the nucleus. Vimentin and β7 integrin were increased to varying degrees. Vitronectin, annexin IV and Ezrin were decreased to varying degrees (Figure 4). These results suggest that Bel-7402-(MRC-5)-CM and MHCC-LM3-(MRC-5)-CM were induced to undergo non-classical EMT via redistribution of α-, β-and γ-catenin, P120 catenin and E-cadherin. Integrins and other adhesion molecules could also be implicated in this induction. These results also reflect dissimilarities in the effect of MRC-5-CM on different cancer cell types. (The fluorescence intensity comparison was showed in Additional files 5, 6: Figure S5–6).

### Effect of MRC-5-CM on Bel-7402 and MHCC-LM3 cell viability

**Figure 5:**
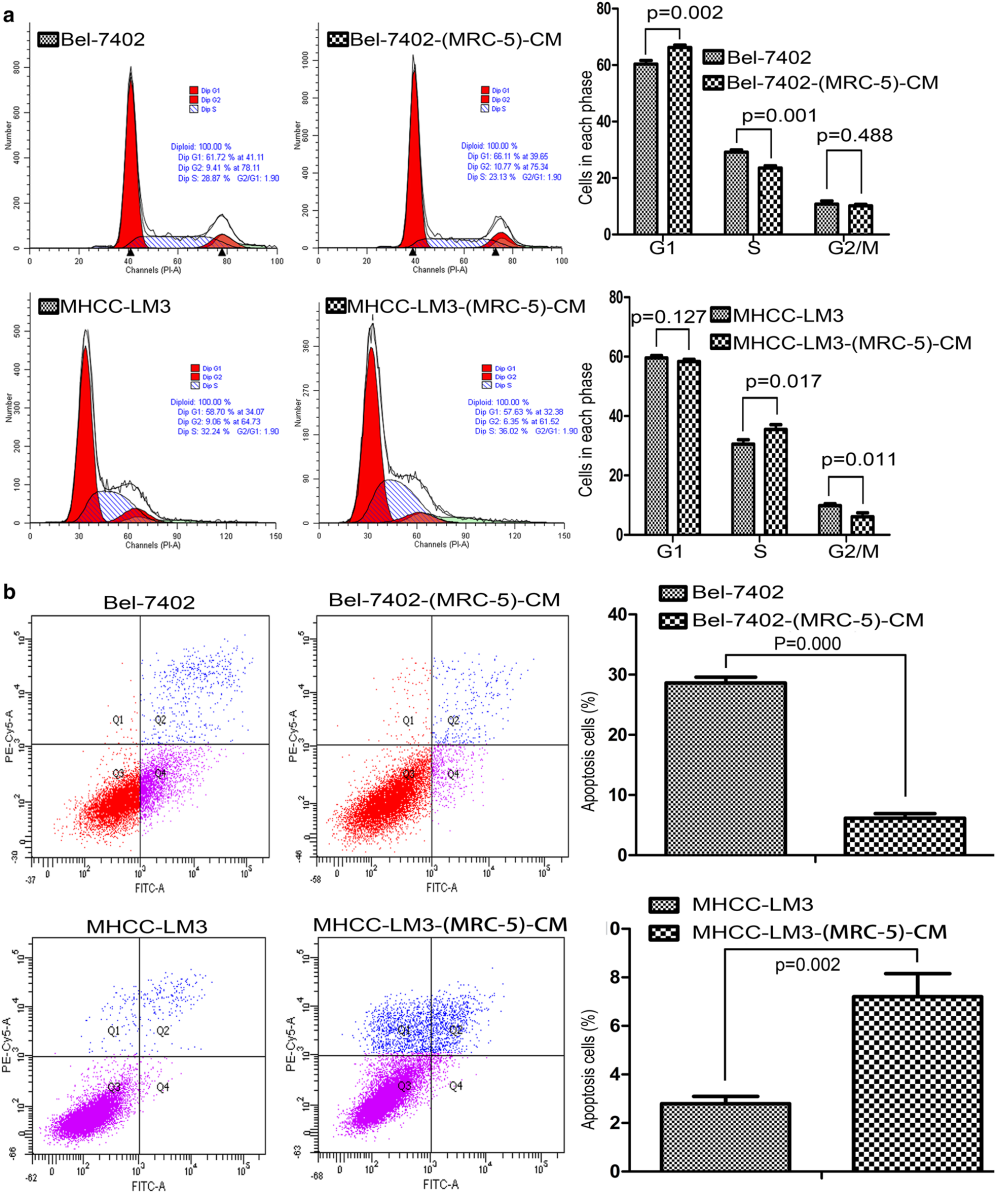
Analysis of cell cycle progression and apoptosis. **a** Cell cycle profiles of Bel-7402 and MHCC-LM3 cells after culture in MRC-5-CM for 21 days were evaluated by flow cytometry. **b** Apoptotic rate of Bel-7402 and MHCC-LM3 cells after culture in MRC-5-CM for 21 days.

To explore the effect MRC-5-CM on tumor cell growth, flow cytometry was used to investigate cell cycle progression and apoptosis. We found that MRC-5-CM induced G1 phase arrest in Bel-7402 cells, while simultaneously inducing S phase arrest in MHCC-LM3 cells (Figure 5a). We also found that MRC-5-CM inhibited apoptosis in Bel-7402 cells while inducing apoptosis in MHCC-LM3 cells (Figure 5b), further suggesting variations in the effect of MRC-5-CM on different cancer cell types.

**Figure 6:**
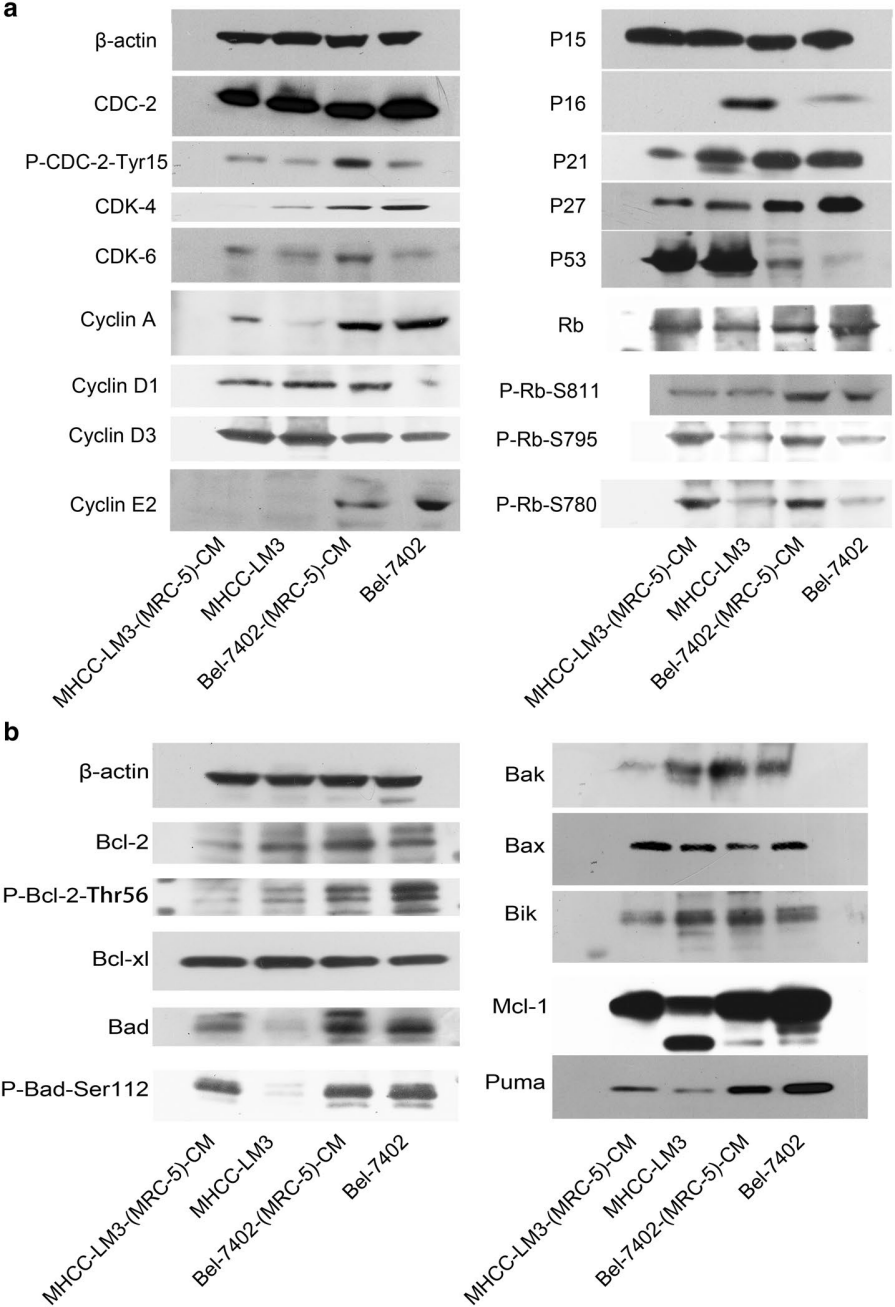
Expression profiles of cell cycle and apoptosis-associated proteins.** a** The expression profiles of cell cycle-associated proteins.** b** The expression profiles of apoptosis-associated proteins

To better understand the effect of MRC-5-CM on Bel-7402 and MHCC-LM3 cell viability, we evaluated the expression of cell cycle- and apoptosis-related proteins. CDK-4, which is essential for the transition from G1 to S phase, was reduced in Bel-7402-(MRC-5)-CM compared with Bel-7402 cells. Moreover, P16 and P53, which inhibit cells in G1 phase from entering S phase, were up-regulated. However, cyclin D1, CDK-6 and phosphorylated Rb (P-Rb-S811, P-Rb-S795 and P-Rb-S780), which promote G1-S phase progression, were increased. Furthermore, P27, which can impede G1-S transition, were unexpectedly down-regulated. Meanwhile, cyclins A and E2, which accelerate the shift into S phase, were downregulated. Cdc-2, which permits the entry of cells from G2 phase into M phase, was decreased, but Tyr15-phosphorylated Cdc-2 (results in Cdc-2 inhibition), was increased. P15, P21, Rb and Cyclin D3 was not changed significantly in Bel-7402-(MRC-5)-CM (Figure 6a). These results also showed that CDK-4 was reduced in MHCC-LM3-(MRC-5)-CM compared with MHCC-LM3. In contrast, Cyclin A, CDK-6 and phosphorylated Rb (P-Rb-S795 and P-Rb-S780) were up-regulated. Moreover, P21 and P27, which halt cell cycle progression were reduced. Cyclins D1, D3, P15, P53, and Rb and P-Rb-S811 did not differ significantly. Cdc-2 was decreased, but Tyr15-phosphorylated Cdc-2 was increased. We were unable to detect the altered expression of Cyclin E2 or P16 in MHCC-LM3-(MRC-5)-CM using equal amounts of lysates (50 μg protein), indicating that basal expressions was minimal (Figure 6a). On the basis of these results, MRC-5 induced G1 phase arrest in Bel-7402 cells and S phase arrest in MHCC-LM3 cells may not be mediated by cyclin, CDK, CKI, P53, and Rb regulation.

The expression profiles of apoptosis-associated genes were also evaluated (Figure 6b). The anti-apoptotic Bcl-2 gene was increased in Bel-7402-(MRC-5)-CM but reduced in MHCC-LM3-(MRC-5)-CM relative to the control. However, the anti-apoptotic Mcl-1 gene was reduced in Bel-7402-(MRC-5)-CM, but increased in MHCC-LM3-(MRC-5)-CM. Thr56-phosphorylated Bcl-2 (interferes with the anti-apoptotic activity of Bcl-2) was reduced in both MHCC-LM3-(MRC-5)-CM and in Bel-7402-(MRC-5)-CM. The anti-apoptotic Bcl-xl gene remained unchanged. Pro-apoptotic genes Bax and Puma were downregulated in Bel-7402-(MRC-5)-CM, but upregulated in MHCC-LM3-(MRC-5)-CM. The pro-apoptotic Bad gene and phosphorylated Bad (inhibits the apoptotic activity of Bad) did not change significantly in Bel-7402-(MRC-5)-CM, but increased in MHCC-LM3-(MRC-5)-CM. Anti-apoptotic genes Bak and Bik were increased in Bel-7402-(MRC-5)-CM, but reduced in MHCC-LM3-(MRC-5)-CM. In view of this, we speculate that the effect of MRC-5-CM on cancer cell apoptosis is mediated partly through regulation of the Bcl-2 gene family, but these genes may not play an essential role.

## Discussion

HCC is prone to rapid infiltration and early diffuse metastases [6]. Because of these pathologic features, the majority of HCC patients are not diagnosed until an advanced stage [17], thus reducing the chance of curative treatment. Survival improvement in patients with late-stage disease requires a better understanding of the regulatory mechanisms of HCC invasion and migration. However, these exact mechanisms are likely multi-factorial and largely unknown [18]. Remarkably, the EMT has been broadly recognized as a critical step in HCC progression [19]. EMT is a dedifferentiation program by which epithelial cells lose cell-to-cell contact and concomitantly gain mesenchymal characteristics, including invasive and migratory abilities. Loss or disruption of tight junctions and E-cadherin/β-catenin complexes at cell boundaries via up-regulation of E-box repressors such as ZEB-1, ZEB-2, Snail, and Twist1 is one of the hallmarks of the EMT. However, recent studies have revealed that such classical EMT events may be the exception [20]. Many normal epithelia migrate efficiently while maintaining integrity, although apico-basal polarity is reduced and tight junctions decreased. Furthermore, branching morphogenesis of the Drosophila tracheal system was associated with the migration of epithelial tubes with enriched E-cadherin and ZO-1 [21]. Interestingly, these results do not prove that EMT is not involved in said movements. In fact, the various modes of cell movement are complex and include the amoeboid (blebbly), amoeboid (pseudopodal, filopodia), mesenchymal, multicellular streaming, and collective migration modes, some of which are interconvertible [22]. The mesenchymal migration mode is characterized by cell–matrix adhesions that become focalized. In addition, the cell forms an elongated spindle-shaped morphology with increased cytoskeletal contractility [23]. Integrins mediate the force generation for the mesenchymal migration.

Our study demonstrated that Bel-7402-(MRC-5)-CM and MHCC-LM3-(MRC-5)-CM displayed mesenchymal characteristics, including an elongated, spread-out morphology, the presence of highly dynamic cellular protrusions and enhanced migration and invasion potentials. We hypothesized that Bel-7402-(MRC-5)-CM and MHCC-LM3-(MRC-5)-CM were induced to undergo non-classical EMT. As the results reveal, the mesenchymal marker vimentin was increased in both cell types. In contrast, the E-cadherin/β-catenin complex, as well as α-, and γ-catenin and P120 catenin were all decreased at the cell surface. Membrane expression of N-cadherin (which mediates cell–cell adhesion in stromal cells) was increased and localized in a disorderly manner on the cell membrane. Classical EMT-promoting transcription factors Snail, Twist1, ZEB-1 and ZEB-2 lost their corresponding roles. Epithelial markers ZO-1 and E-cadherin were also increased. Interestingly, we found a novel phenomenon in which γ-catenin was more inclined to be expressed in the nucleus. It is worth noting that induction of the non-classical EMT may be mainly via redistribution of α-, β-, and γ-catenin, P120 catenin, E-cadherin and N-cadherin.

Laminins, the prominent components of basement membranes, are large heterotrimeric glycoproteins composed of one α, one β, and one γ chain. Integrins, a family of cell surface adhesion receptors, recognize different extracellular matrix components and are comprised of one α and one β subunit. Laminins and integrins are intimately related to cancer progression via interaction with MMPs. Previous studies revealed that laminins A1 and B3 can promote the malignant phenotype of melanoma and non-small cell lung cancer [24, 25], and α6, β1, β3, β4 and β7 integrins were essential for cancerous invasion [26–30]. The interaction between MMPs and laminins/integrins has received ample attention, and MMP-2 is believed to be one of the major proteases involved in mesenchymal migration [31–33]. Therefore, we evaluated the expression of Laminin A1, Laminin B3,as well as α6, β1, β3, β4 and β7 integrins, FAK, P-FAK-Y397, Src, P-Src-Y529 and MMPs in both Bel-7402-(MRC-5)-CM and MHCC-LM3-(MRC-5)-CM. In agreement with previous studies, α6, β3, β4 and β7 integrins were up-regulated in both cell types. Laminin A1 was upregulated in Bel-7402-(MRC-5)-CM while Laminin B3 was up-regulated in MHCC-LM3-(MRC-5)-CM. Moreover, FAK was activated in both cell types in response to increased integrin expression. Nevertheless, β1 integrin remained unchanged. The expression profiles of MMPs indicated that MMP-2 and -3 were up-regulated in Bel-7402-(MRC-5)-CM, and that MMP-14, -17 and MMP-2 were increased in MHCC-LM3-(MRC-5)-CM. On the basis of these results, we speculated that Bel-7402-(MRC-5)-CM and MHCC-LM3-(MRC-5)-CM were promoted to invade and migrate, possibly through activation of FAK and upregulation of MMP-2. Other adhesion molecules, including Vitronectin, Annexin IV, and Ezrin, may also be implicated in this mechanism through their interactions with laminins/integrins.

Our previous study verified that MRC-5-CM inhibited the mRNA level of β4 integrin in Bel-7402 cells. We speculate that MRC-5-CM enables β4 integrin to avoid the natural degradation at the post transcriptional level by translocation. Thus, β4 integrin could activate downstream signal molecules consistently. We also have mentioned that β4 integrin is negatively correlated with CK19 expression (which is a valuable predictor of HCC recurrence). It reflects that HCC cells acquire a large amount of β4 integrin just at the stage of the metastatic process. Therefore, the role of β4 integrin in HCC remains to be further explored.

In the present study, we demonstrated that MRC-CM mediates both cell cycle progression and apoptosis. We found that MRC-5-CM induced G1 phase arrest in Bel-7402 and S phase arrest in MHCC-LM3 cells. We also found that MRC-5-CM inhibited apoptosis in Bel-7402 while MRC-5-CM induced apoptosis in MHCC-LM3 cells. The cell cycle is regulated mainly by cyclins, CDKs and CKIs [34–36] whereas apoptosis is regulated mainly by the Bcl-2 family of genes [37–39]. We examined the expression profiles of cyclins, CDKs, CKIs, P53, Rb, phosphorylated Rb, pro-apoptotic Bcl-2 family proteins (Bad, Bax, Bik, Bak and Puma) and pro-survival Bcl-2 family proteins (Bcl-2, Bcl-xL and Mcl-1) in Bel-7402-(MRC-5)-CM and MHCC-LM3-(MRC-5)-CM. The results indicated that typical cell cycle-related proteins and cell apoptosis-related proteins (such as those mentioned above) did not play a key role in the MRC-5-CM model, and another novel mechanism mediating cancer cell proliferation and apoptosis must be present.

## Conclusion

MRC-5-CM mediated multiple pathways regulating both proliferation and apoptosis in HCC cells. MRC-5-CM facilitated HCC cell invasion and migration through three mechanisms: redistribution of α-, β- and γ-catenin, P120 catenin, E-cadherin and N-cadherin, activation of the integrin/FAK/Src signaling pathway and upregulation of MMP2. We are not certain that the decreases in E-cadherin/β-catenin complexes, as well as α-catenin, γ-catenin and P120 catenin at the cell surface were due to the increased integrin expression, but we do know that α9β1 integrin can form a tri-partite protein complex with β-catenin and E-cadherin. Thus, we are conducting further study to explore the role of α6, β3, β4 and β7 integrins in HCC.

## Additional files

Additional file 1:
**Figure S1.** Ratio discrepancy of the target proteins/β-actin between HCC cells cultured in MRC-5-CM and HCC cells. Additional file 2:
**Figure S2.** Ratio discrepancy of the target proteins/β-actin between HCC cells cultured in MRC-5-CM and HCC cells. Additional file 3:
**Figure** **S3.** Ratio discrepancy of the target proteins/β-actin between HCC cells cultured in MRC-5-CM and HCC cells. Additional file 4:
**Figure S4.** Ratio discrepancy of the target proteins/β-actin between HCC cells cultured in MRC-5-CM and HCC cells. Additional file 5:
**Figure S5.** Fluorescence intensity comparison of EMT-related proteins between HCC cells cultured in MRC-5-CM and HCC cells. Additional file 6:
**Figure** **S6.** Fluorescence intensity comparison of EMT-related proteins between HCC cells cultured in MRC-5-CM and HCC cells.
