# Supplementary figures and images for: MRC-5 fibroblast-conditioned medium influences multiple pathways regulating invasion, migration, proliferation, and apoptosis in hepatocellular carcinoma
Source: J Transl Med. 2015 Jul 22;13:237. doi: 10.1186/s12967-015-0588-8 (PMC4508812; doi:10.1186/s12967-015-0588-8)

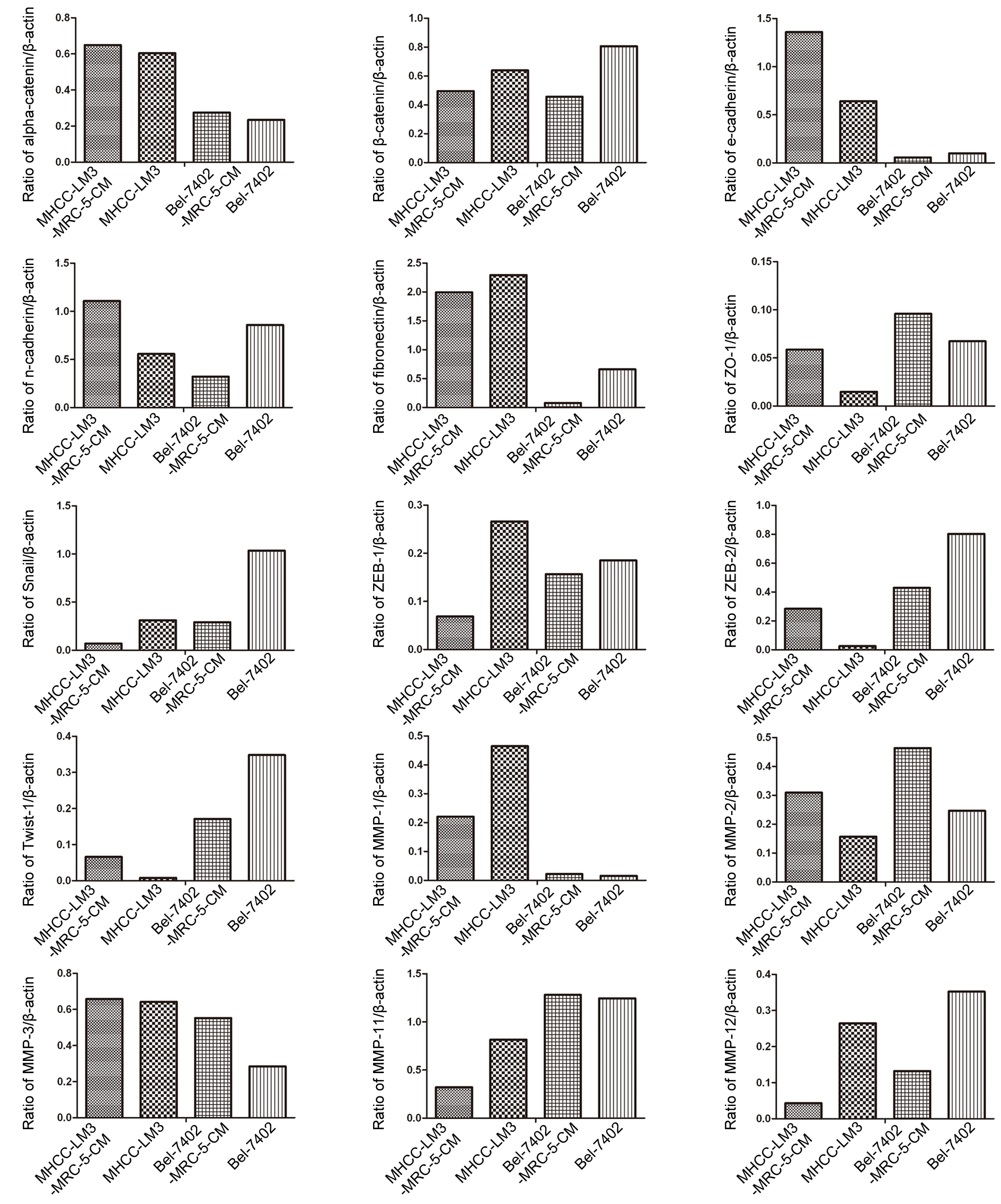

Supplement: Additional file 1: — Figure S1. Ratio discrepancy of the target proteins/β-actin between HCC cells cultured in MRC-5-CM and HCC cells. [file 12967_2015_588_MOESM1_ESM.tiff]

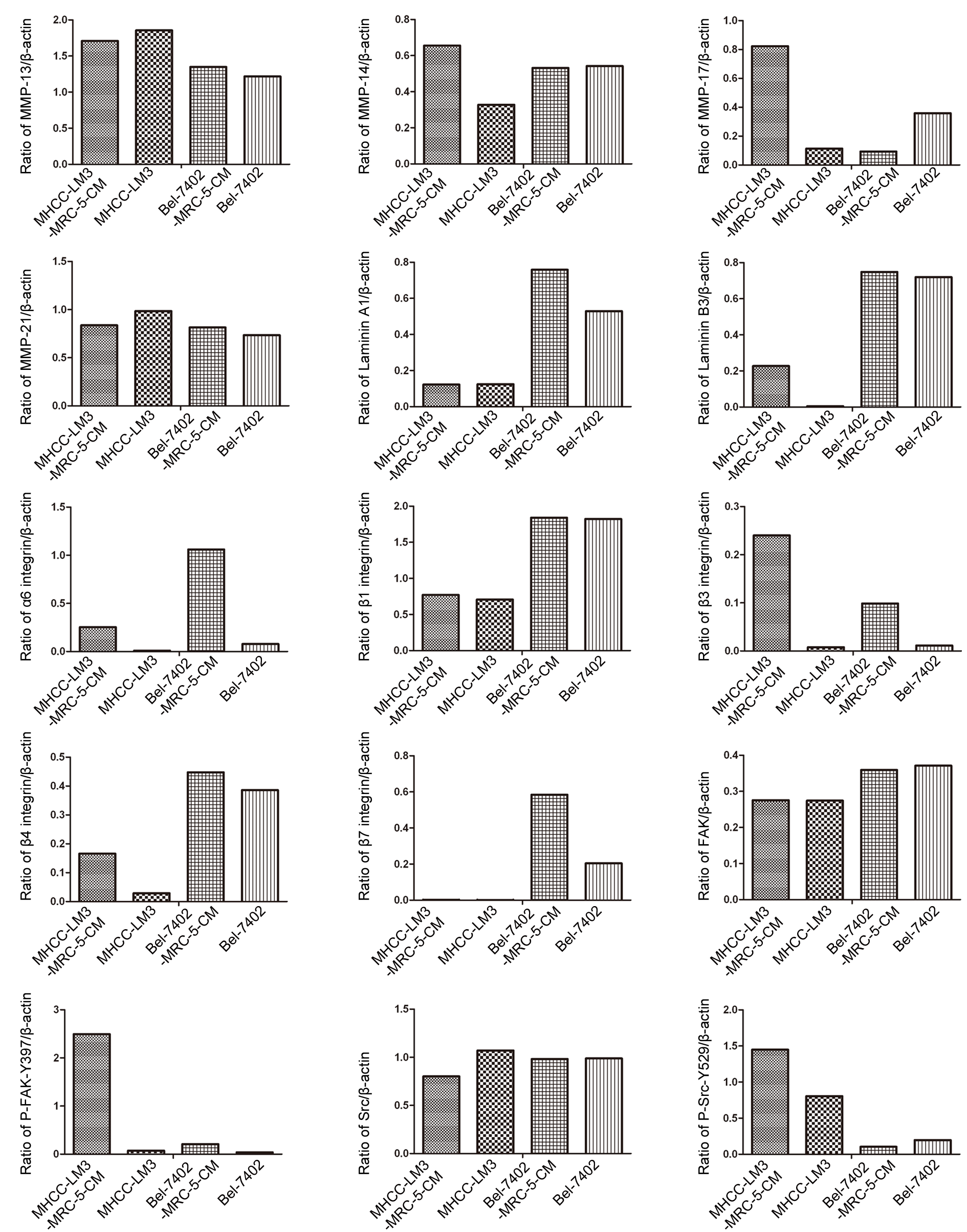

Supplement: Additional file 2: — Figure S2. Ratio discrepancy of the target proteins/β-actin between HCC cells cultured in MRC-5-CM and HCC cells. [file 12967_2015_588_MOESM2_ESM.tiff]

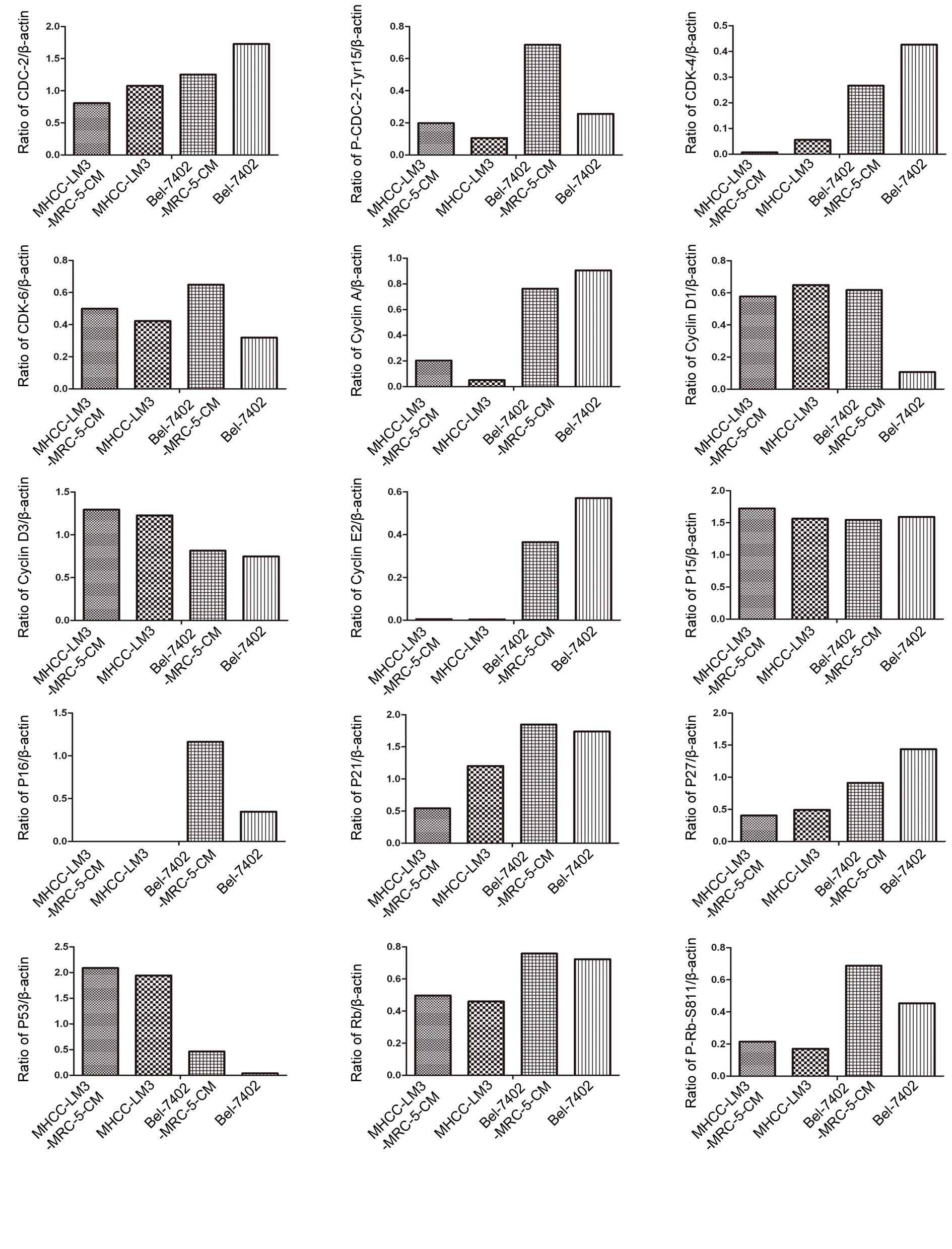

Supplement: Additional file 3: — Figure S3. Ratio discrepancy of the target proteins/β-actin between HCC cells cultured in MRC-5-CM and HCC cells. [file 12967_2015_588_MOESM3_ESM.tiff]

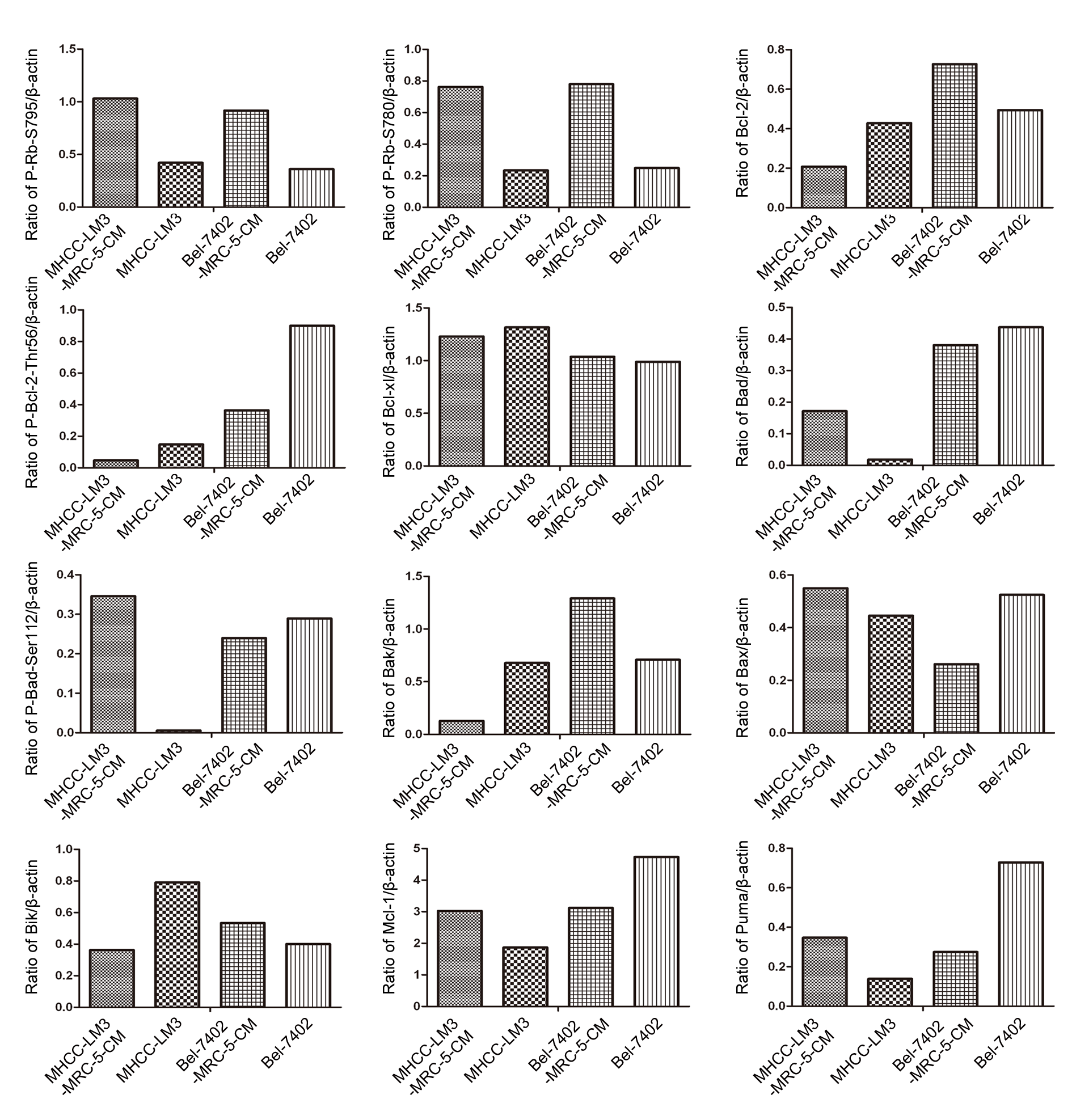

Supplement: Additional file 4: — Figure S4. Ratio discrepancy of the target proteins/β-actin between HCC cells cultured in MRC-5-CM and HCC cells. [file 12967_2015_588_MOESM4_ESM.tiff]

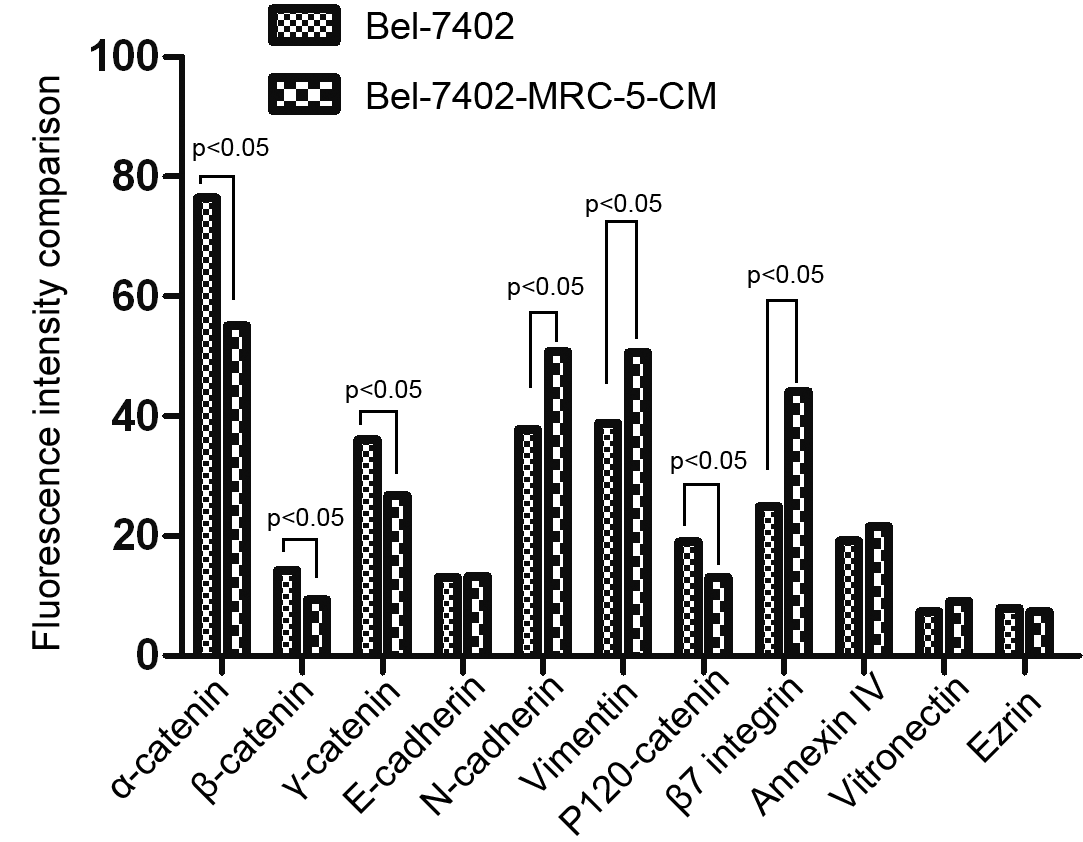

Supplement: Additional file 5: — Figure S5. Fluorescence intensity comparison of EMT-related proteins between HCC cells cultured in MRC-5-CM and HCC cells. [file 12967_2015_588_MOESM5_ESM.tiff]

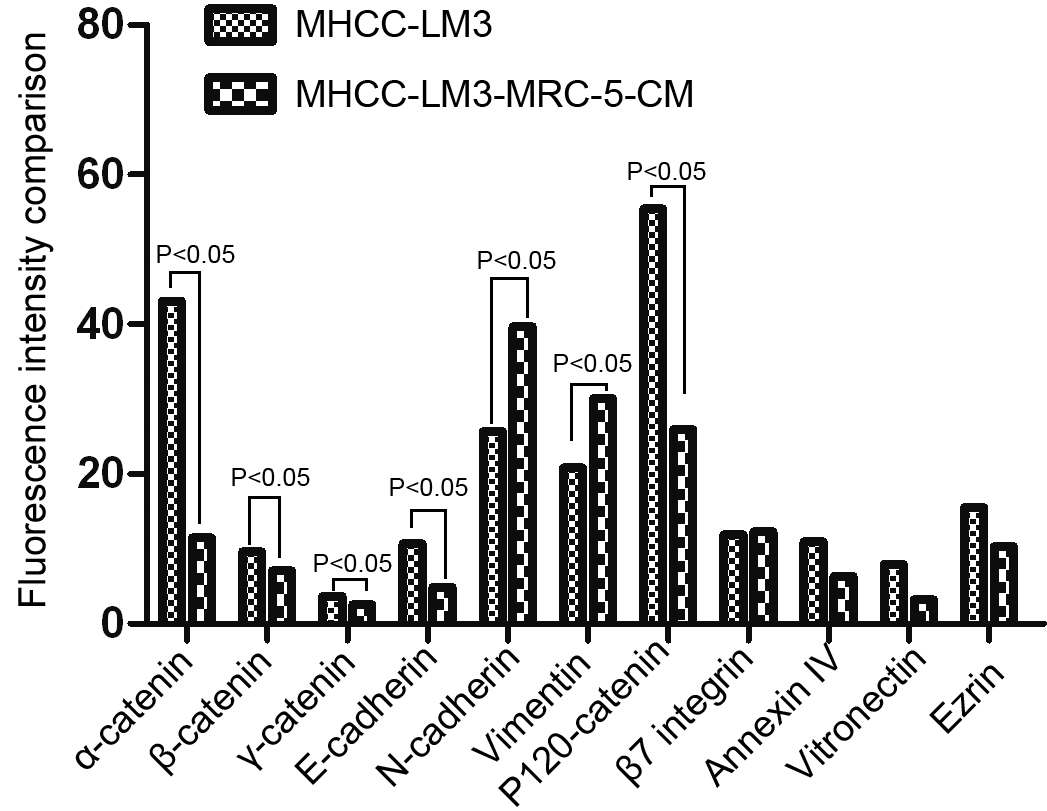

Supplement: Additional file 6: — Figure S6. Fluorescence intensity comparison of EMT-related proteins between HCC cells cultured in MRC-5-CM and HCC cells. [file 12967_2015_588_MOESM6_ESM.tiff]
